# Transparency and reproducibility in evolutionary research

**DOI:** 10.1002/ece3.2291

**Published:** 2016-07-19

**Authors:** Ruth G. Shaw, Allen J. Moore, Mohamed Noor, Michael G. Ritchie

The vitality of the science of evolutionary biology depends on new research findings that continually advance understanding, and the sound approaches by which those findings are obtained must be clearly documented. This transparency of documentation crucially grounds others' assessment of the credibility of the inferences and interpretations. It is also essential to attempts of future scientists, in conducting independent research, to validate the findings and assess their generality, advancing the field still further. These core aspects of science are widely appreciated. Even so, there is increasing awareness that publication practice sometimes falls well short of the goal of transparency that supports a thorough evaluation of the results, sufficient information for research synthesis, and the possibility of replicating of a study.

As editors of journals that publish research in evolutionary biology, three of us (MR, AM, and RS) recently participated in a workshop (Improving Inference in Evolutionary Biology and Ecology, Nov 12‐13 2015, Center for Open Science, Charlottesville VA). This workshop heightened our awareness of problems of publication practice that too frequently compromise transparency and, consequently, impair understanding and can give a false impression of reliability. In an effort to alleviate these problems and thereby enhance scientific progress, Nosek et al. (2015) have laid out general guidelines for research reporting as the Transparency and Openness Promotion (TOP) framework (https://cos.io/top/) comprising eight specific recommendations (TOP1‐8). Recommendations for the implementation of these general guidelines in the fields of evolutionary biology and ecology have been proposed as Tools for Transparency in Ecology and Evolution (TTEE): (https://osf.io/g65cb/wiki/home/).

We fully support the goals of openness and transparency, and our journals' current policies align with the TOP to a great degree. In particular, the requirement to publicly archive all data on which an article is based, in effect since 2011, is crucially important in supporting these goals, as is the requirement to cite datasets that are already in the public domain. These policies address the first set of TOP guidelines (TOP1‐2). The third guideline (TOP3) concerns archiving of computer code. Currently, this is required for simulation studies, although not for analyses of data. We applaud the trend that, increasingly, authors archive their analysis code along with their data, and we advocate that this practice, and transparency of research material (TOP4) be adopted even more widely.

We also expect that articles clearly lay out details of empirical methods and of data analysis and that they thoroughly, openly report results (TOP 5). Reviewers', editors', and eventual readers' evaluation of how reliable a study is may depend critically on these specifics, which are also the basis for future efforts to build on the research. Because evolutionary and ecological research is wide‐ranging, spanning theory, and empirical studies, which may be observational or experimental, no single list can easily capture what should be reported, but the TOP (pp. 2–4) provides valuable guidance for authors, as well as for those involved in publication decisions.

Preregistration of studies (TOP6) and research plans (TOP7) is not currently a requirement of journals in evolutionary biology. Our view is that it should remain optional, but we agree that the TOP framework makes a strong case that, for example, distinguishing preplanned and *post hoc* analyses can greatly aid the reader to understand and assess the context of many analyses.

The final recommendation (TOP8) concerns replication studies. Creative development of new lines of inquiry and new approaches to addressing important and recalcitrant questions in evolutionary biology are emblematic of vitality of a science. Nevertheless, as evolutionary biologists, we strive through our research to make evolutionary advances of general import. This demands a record of the research specifics as a foundation for assessing that generality via replication of published studies. We recognize that precise replication is an ideal that is not available for much of the research in evolutionary biology, given that many studies concern unique populations in unique contexts, as is especially apparent for research conducted in nature. Moreover, severe limitations of research funding make support for replication less likely. Yet, as we have noted, advance of a science depends on rigorous, independent research to validate and assess the generality of previously reported results. Because of the critical importance of this aspect of scientific inquiry, our journals will continue to publish sound research designed to assess the validity or generality of findings from previously reported empirical studies addressing compelling, unresolved questions, alongside studies that justify a claim of novelty. We note that the judgment of whether a particular finding has been “replicated” too often hinges on whether the new study aligns with the previous one in rejecting the null hypothesis. Coherent insight will not emerge from research outcomes so simplistically integrated. Rather, magnitudes of biological effects, along with their uncertainty due to inevitable sampling variation, are critically important to advancing evolutionary understanding. Consideration that differences in findings may arise from difference in context is also imperative.

Our journals' policies promote transparency, but cannot guarantee it. In the interest of the broad scientific and social benefits that result, we urge authors to redouble their efforts to make their reports of their research methods and results as clear and open as possible.

